# Standardised classification of pre-release development in male-brooding pipefish, seahorses, and seadragons (Family Syngnathidae)

**DOI:** 10.1186/1471-213X-12-39

**Published:** 2012-12-29

**Authors:** Stefan Sommer, Camilla M Whittington, Anthony B Wilson

**Affiliations:** 1 Institute of Evolutionary Biology and Environmental Studies, University of Zürich, Winterthurerstrasse 190, Zürich, CH-8057, Switzerland

**Keywords:** Developmental stage, Embryo, *Hippocampus*, Larva, *Nerophis*, *Syngnathus*

## Abstract

**Background:**

Members of the family Syngnathidae share a unique reproductive mode termed male pregnancy. Males carry eggs in specialised brooding structures for several weeks and release free-swimming offspring. Here we describe a systematic investigation of pre-release development in syngnathid fishes, reviewing available data for 17 species distributed across the family. This work is complemented by in-depth examinations of the straight-nosed pipefish *Nerophis ophidion*, the black-striped pipefish *Syngnathus abaster*, and the potbellied seahorse *Hippocampus abdominalis*.

**Results:**

We propose a standardised classification of early syngnathid development that extends from the activation of the egg to the release of newborn. The classification consists of four developmental periods – early embryogenesis, eye development, snout formation, and juvenile – which are further divided into 11 stages. Stages are characterised by morphological traits that are easily visible in live and preserved specimens using incident-light microscopy.

**Conclusions:**

Our classification is derived from examinations of species representing the full range of brooding-structure complexity found in the Syngnathidae, including tail-brooding as well as trunk-brooding species, which represent independent evolutionary lineages. We chose conspicuous common traits as diagnostic features of stages to allow for rapid and consistent staging of embryos and larvae across the entire family. In view of the growing interest in the biology of the Syngnathidae, we believe that the classification proposed here will prove useful for a wide range of studies on the unique reproductive biology of these male-brooding fish.

## Background

Pipefish, seahorses, and seadragons (Syngnathidae) are teleost fish that occur in tropical, subtropical, and temperate environments around the world [1]. To date approximately 300 extant species are known [2], most of which live in near-shore marine habitats, although some species live in the open sea or in brackish or fresh-water [1]. Increased interest in the biology of these fish in recent years has led to the development of syngnathids as model organisms for studies in evolution, ecology, and conservation biology [3], with particular attention being directed towards their extraordinary reproductive biology.

Common to all syngnathids is a unique reproductive system known as male pregnancy [4]. At the end of species-specific courtship dances [5] females transfer oocytes to specialised brooding structures located on the ventral side of either the trunk (Gastrophori) or the tail (Urophori) of the males’ body. These structures vary considerably between species, ranging from open brooding areas (Nerophinae) to marsupia with protective pouch flaps (Syngnathinae) to sealed pouches (Hippocampinae) [6,7]. In all species, males brood fertilised eggs for extended periods and release independent and free-swimming offspring (e.g. [8-11]).

Embryonic and larval development have been described for a number of syngnathid species including pipefish [10,12-15], seahorses [9,11,12,16], and seadragons [17]. However, to date no attempt has been made to standardise early developmental processes for all members of the family. Given the number of comparative studies on syngnathid reproductive biology (e.g. [18-24]), and in view of the growing scientific interest in these fish, such a standardisation is essential for cross-species comparisons. In the present account, we describe common features in the development of syngnathids, reviewing available data (lit. cit. above) and examining staged specimens of the straight-nosed pipefish *Nerophis ophidion* (a trunk-brooding species with an open marsupium), the black-striped pipefish *Syngnathus abaster* (a tail-brooding species with fused pouch flaps), and the potbellied seahorse *Hippocampus abdominalis* (a tail-brooding species with a sealed pouch) [6].

## Results

**Figure 1:**
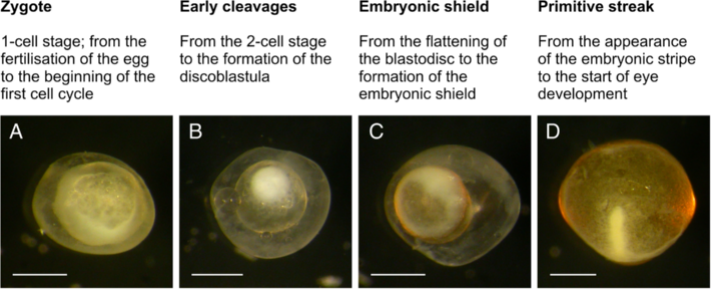
**Early embryogenesis.** Descriptions of the four stages of the early-embryogenesis period, along with examples for each stage. (**A**) Animal-pole view of a zygote of *N. ophidion* ca. 45 min after mating. (**B**) Animal-pole view of a *N. ophidion* blastula during early cleavages. (**C**) Embryonic-shield stage in *N. ophidion*; the white circle represents the germ ring. (**D**) Primitive-streak embryo of *S. abaster* (dechorionated). Scale bars are 0.5 mm.

**Figure 2:**
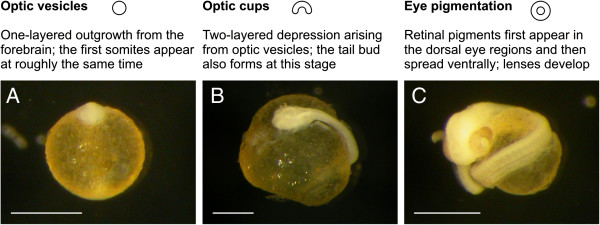
**Eye development.** Descriptions and schematic drawings of stage-defining eye-structures of the three stages of the eye-development period, along with examples for each stage. (**A**) Optic-vesicle stage in *N. ophidion*. (**B**) Optic-cup stage in *S. abaster*. (**C**) Eye-pigmentation stage in *N. ophidion*. All embryos were dechorionated prior to photographing. Scale bars are 0.5 mm.

**Figure 3:**
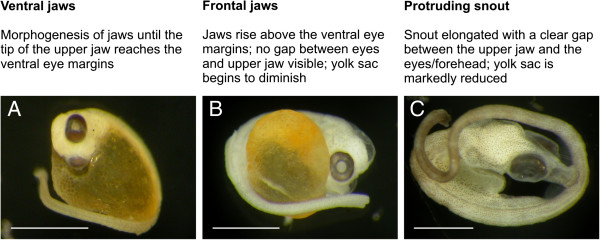
**Snout formation.** Descriptions of the three stages of the snout-formation period, along with examples for each stage. (**A**) *S. abaster* embryo (dechorionated) with ventrally developing jaws. (**B**) *H. abdominalis* larva with jaws rising frontally. (**C**) *S. abaster* larva with an elongated snout. Scale bars are 1 mm.

**Figure 4:**
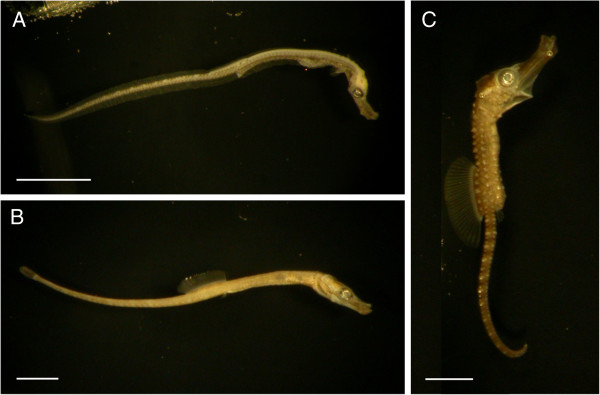
**Newborns of three syngnathid species.** The newborn stage represents the first stage of the juvenile period and, for the purpose of this classification, refers to the first day post-release. Shown are (**A**) *N. ophidion*, (**B**) *S. abaster*, and (**C**) *H. abdominalis*. Scale bars are 2 mm.

We distinguish four developmental periods in early syngnathid development composed of 11 stages (Figures 1, 2, 3, 4), characterised by the appearance (or disappearance, in the case of the yolk sac) of external morphological traits. Naturally, these stages are not specific time points, but rather reflect discrete periods that subdivide continuous development into a sequence of distinct units (cp. [25]). Our description starts with the activation of the egg and ends upon the release of free-living juveniles.

### Early embryogenesis

During the first period we distinguish four stages: zygote, early cleavages, embryonic shield, and primitive streak (Figure 1). Individual cell cycles can be further partitioned for studies aimed at investigating early development (cp. [11,13,26]). The first two and the last two stages roughly coincide with blastulation and gastrulation, respectively.

The initial stages in the embryogenesis of syngnathid fishes follow the pattern common to all teleosts [27]. Upon activation of the egg (zygote stage; Figure 1A) the cytoplasm accumulates at the animal pole. The first few cleavages are synchronous – in *Syngnathus acusimilis* up to the 64-cell stage [13] – and meroblastic (incomplete); they produce blastomeres of roughly equal size [10,11,13] (but see [26]). Further divisions lead to the formation of the discoblastula [9], which is characterised by the blastoderm resting on top of the uncleaved yolk mass (Figure 1B). The blastoderm then flattens and moves towards the vegetal pole [13,15]), marking the onset of epiboly (the enclosing of the yolk by the blastoderm). Around the time the blastoderm margin (germ ring) passes the equator, cells accumulate at a specific position along the germ ring to form the embryonic shield (Figure 1C). At this stage of development, the dorsoventral and anteroposterior axes of the developing embryo can be identified – the shield designates what will become the dorsal region of the embryo, while cells located towards the animal pole will develop into the head region (cp. [25]). At a more advanced stage of epiboly, the embryo emerges as a primitive streak on top of the yolk (Figure 1D).

### Eye development

Eyes are the first conspicuous trait to develop in the primitive-streak embryo [17]; somites become faintly visible at roughly the same time [11,16]. We distinguish three stages of eye development: optic vesicles, optic cups, and eye pigmentation (Figure 2).

Optic vesicles appear towards the end of epiboly as semi-spherical structures parallel to the forebrain (Figure 2A; cp. [15]). At around the time these structures develop into optic cups (Figure 2B), the tail bud develops. Optic cups are banana-shaped, two-layered eye structures, with the inner and outer layer forming the neural and the pigmented retina, respectively (cp. [25]). Retinal pigments first appear in the dorsal region of the eyes and then start spreading ventrally (Figure 2C) [16]; the first body pigments become visible at roughly the same time [9,15,16]. As the embryo elongates, the tail gradually detaches from the yolk sac [13,14,16,22,27]. Embryos of species with closed marsupia (e.g., *Hippocampus*, *Syngnathus*) hatch around this stage but are retained within the pouch, thus entering the larval phase (cp. [25]), while embryos of species with open brooding structures (e.g., *Nerophis*, *Phyllopteryx*) continue to develop inside the egg shell [9-11,13,15,17]. At this stage of development both embryos and larvae are still feeding on their yolk reserves.

### Snout formation

Syngnathids are suction-feeding fish characterised by tubular snouts composed of elongated, fused jaws [1]. During snout formation, the yolk sac gradually disappears [10,11,13,15,16]. Many species- or group-specific traits start developing during this period (e.g., body colouration, fleshy appendages in seadragons, and prehensile tails in seahorses). However, such traits are not appropriate as diagnostic features in a family-wide classification of developmental processes. For the purpose of this classification, we distinguish three stages of snout formation: ventral jaws, frontal jaws, and protruding snout (Figure 3).

Jaw formation starts before the eyes are fully pigmented. Jaws develop ventral to the eyes (Figure 3A) and first grow horizontally to the dorsoventral axis (e.g. [14,16]); later they rise vertically. When the tip of the upper jaw reaches the middle of the eyes, the jaws are still in close contact with the forehead (Figure 3B; e.g. [13,16,17]). The snout then gradually elongates as a result of a lengthening of the ethmoid and the quadrate cartilages [11], approaching the protruding adult form (Figure 3C; cp. [10,14-16]). Snout formation is completed prior to release [9,13].

### Juvenile

Data on post-release development is generally scarce [27] (but see [28]). Here we describe only the first stage (newborn; Figure 4) of the juvenile period, which is the last stage in our description of early syngnathid development.

At release the yolk sac is usually fully resorbed (but see [17]) and juveniles switch to external feeding [8-10]. However, under stressful conditions males may release premature juveniles still carrying yolk sac reserves [10,16]. Many traits continue to develop post-release. Most notable among these are the fins, which differ markedly among species in their pattern of development (for a discussion of pre-release fin development in syngnathids, see Additional file 1), scales and body pigmentation, as well as fleshy appendages in seadragons (e.g. [8,10,17,27]).

## Discussion

This study aims to provide a unified description of early developmental processes for pipefish, seahorses, and seadragons. The classification we propose is derived from tail-brooding and trunk-brooding species distributed across the entire phylogenetic tree (cp. [2]), and is based on species representing a variety of morphologically distinct adult forms, including species with different degrees of brooding-structure complexity [6]. As such, we believe that the classification is likely applicable to the majority of species of this family. Moreover, a comparison with early developmental processes in the Solenostomidae (ghost pipefish), the closest relatives of the Syngnathidae (cp. [2]), suggests that the staging method applied here may also be applicable to embryos and larvae of this family of female brooders.

The Solenostomidae are a small family of skin-brooding, viviparous fish from the Indo-Pacific region [1]. Solenostomid females brood eggs in a pouch built of enlarged pelvic fins, the borders of which are fused to the ventro-lateral sides of their body [29]. Early development has been described for one such species, *Solenostomus cyanopterus*[30], and closely resembles the stages of syngnathid development described here. *S. cyanopterus* larvae hatch inside the pouch around the time the jaws start elongating [30]. Similar to larvae of syngnathid species with closed brooding structures, solenostomid larvae continue to develop inside the marsupium before they are released. At release, the tubular snout is formed and the yolk reserves are exhausted [30].

Given that release from the male’s body entails a change in juvenile lifestyle that is comparable among all syngnathids, we chose newborn as a distinct stage in our classification, despite marked differences between species in the developmental progress of some traits at release. Based on the three species examined in this study, we speculate that developmental differences among species may reflect differences in brood-pouch complexity. Such a relationship between brooding-structure complexity and developmental progress might be expected (cp. [11,15]), as embryos of species with open brooding areas are released at the time of hatching, whereas larvae of pouch-bearing species, once hatched, continue to develop inside the pouch for days or weeks before they are released into the open water.

**Figure 5:**
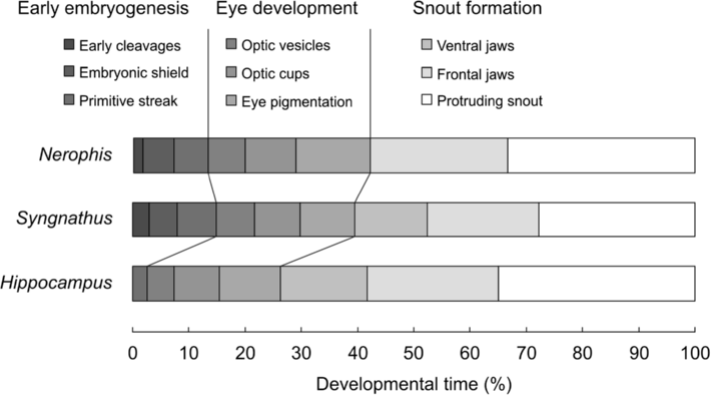
**Approximate relative duration of developmental stages in *Nerophis*, *Syngnathus*, and *Hippocampus*.** Stage lengths between fertilisation (0%) and release (100%) are estimated for *N. lumbriciformis* (average developmental time: 30 days, water temperature: 14-15°C; [10]), *S. abaster* (28 days, 18-19°C; [15]), *H. guttulatus* and *H. brevirostris* (21 days, 23°C; [16]), and *H. kuda* (25 days, 24-29°C; [9]); stage-length estimates for *Hippocampus* are averaged across the two publications. Note that zygote stages (usually a few hours), early cleavages and the embryonic-shield stage in *Hippocampus*, and the ventral-jaws stage in *Nerophis* are not shown, as information on the duration of these stages was not available from the original publications.

We did not attempt to estimate the duration of the proposed stages in absolute terms, because the duration of brooding is negatively correlated with ambient water temperature [10,15,31,32] and, for any given temperature, likely differs among species, particularly between those from tropical and temperate habitats. However, the relative duration of stages can be estimated by converting their lengths into relative contributions to total brooding time, assuming that rates of development at different stages are similarly sensitive to changes in temperature (for zebrafish, see [25]). We estimated the relative length of each developmental stage for three genera – *Nerophis*, *Syngnathus*, and *Hippocampus* – and found that stage lengths increase progressively from activation of the egg to release of the newborn (Figure 5). This is to be expected, as the rate of morphological change is more dramatic in the embryo than at later developmental stages [13,16]. Moreover, ontogenetic trajectories diverge; consequently, fewer traits suitable as diagnostic features for our family-wide classification are available as development progresses.

An alternative method of comparing rates of development between species would be to maintain captive-mated males under identical conditions during brooding and to compare the developmental progress of age-matched individuals. Preliminary examinations of *N. ophidion*, *S. typhle*, and *H. abdominalis* suggest that embryos and larvae of these species develop at approximately the same rate. When held at water temperatures of 18–19°C and a salinity of ca. 35 ppt, embryos of all three species were at the optic-cup or eye-pigmentation stage after 8 days of development and at the frontal-jaw stage after 16 days (CMW and ABW, unpublished data), corresponding to roughly one fourth and one half of total developmental time, respectively (cp. Figure 5).

## Conclusions

In the present account, we propose a straightforward classification of early syngnathid development based on examinations of a wide range of species spread across the syngnathid phylogeny. This classification consists of four periods that are divided into 11 stages. Diagnostic features of stages are morphological traits that are visible in live as well as in preserved material and can be identified without the need for dissection. As such, the proposed classification allows for rapid and consistent staging of pipefish, seahorses, and seadragons. We used names, instead of numbers, to label stages, since a system based on names provides more flexibility [25]. While this classification has the advantage of cross-species applicability, stages can be easily added or removed from this general classification depending on the specific research questions and the species involved. In view of the growing interest in the biology of the Syngnathidae [33], we believe that the classification proposed here will prove useful to a wide range of researchers studying the reproductive biology of this group.

## Methods

Embryos (pre-hatch), larvae (post-hatch/pre-release), and newborn juveniles of *N. ophidion* and *S. abaster* were obtained from wild-caught brooding males from the Venice Lagoon (Italy), fixed in 10% formalin and preserved in 70% ethanol, and from experimentally mated wild-caught males held at our marine husbandry facility at the University of Zürich, Switzerland. Pipefish were collected with permission (no. 39980/2012) from the Servizio Caccia e Pesca, Provincia di Venezia, Italy. Adult seahorses (*H. abdominalis*) were purchased from a commercial breeding facility (Seahorse Australia, Beauty Point, Tasmania); seahorse offspring were derived from males that had mated in Zürich. Both pipefish and seahorses were held under an animal care permit from the Veterinäramt Zürich (Permit 164/2010). Photographs of staged specimens were taken using a digital camera (Nikon Coolpix E4500) connected to a light microscope (Wild M3, Heerbrugg, Switzerland).

## Competing interests

The authors declare that they have no competing interests.

## Authors’ contributions

SS conceived the study, performed the literature review, examined the fish material, and drafted the manuscript. CMW helped in the conception of the study and the drafting of the manuscript. ABW collected the fish, assisted in the design of the study, and participated in drafting the manuscript. All authors read and approved the final manuscript.

## Supplementary Material

Additional file 1 **Fin development.** A discussion of syngnathid fin development, which shows considerable variation among species. Click here for file
