# Supplementary material for: Standardised classification of pre-release development in male-brooding pipefish, seahorses, and seadragons (Family Syngnathidae)
Source: BMC Dev Biol. 2012 Dec 29;12:39. doi: 10.1186/1471-213X-12-39 (PMC3541971; doi:10.1186/1471-213X-12-39)
Supplement: Additional file 1 — Fin development. A discussion of syngnathid fin development, which shows considerable variation among species. [file 1471-213X-12-39-S1.pdf]

Additional file 1

Fin development

Adult syngnathids may have a single dorsal, caudal, and anal fin, as well as a pair of pectoral fins [1]. However, not all species develop all four types of fins. Moreover, the developmental progress of fins varies considerably between offspring of different species (Table A1). Here we distinguish three stages of fin development: fin bud, fin blade, and fin rays.

In seahorses (genus *Hippocampus*), pectoral and dorsal fin buds form first, around the optic-cup stage [16], while the anal fin bud develops shortly after the onset of eye pigmentation [11]. Pipefish show considerable species-level variation in fin development, and pectoral (*Microphis leiaspis* [28]; *S. abaster*, this study), caudal (*Micrognathus brevirostris* [34]) or dorsal (*Syngnathoides biaculeatus* [14]) fin buds may form first, usually during early eye development; the anal fin bud generally appears last.

Fin blades tend to develop in the same species-specific order as the buds. In seahorses and seadragons they are formed before the snout begins to elongate [16,17]. In certain pipefish species some fin blades develop only after release (cp. Table A1).

Fin rays develop in a different order than the buds and blades. Generally, they first appear in the caudal and the dorsal fin, then in the pectoral fins, and last in the anal fin [13,34-36] (this study). In seahorses, which lack caudal fins, rays first develop in the dorsal fin. According to Boisseau [16] and Wetzel and Wourms [11], fin rays then appear in the pectoral fins and last in the anal fin, whereas the sketches of Leiner [12] suggest that rays appear last in the pectoral fins. All fin rays are formed prior to release in the seahorse [9,11,16]. In *S. biaculeatus*, a pipefish species without a caudal fin [37], dorsal fin rays develop first, before the snout begins to elongate, whereas anal and pectoral fin rays appear afterwards; all rays are formed before the snout is fully elongated [14].

Nerophis pipefish are a somewhat special case, as embryos of this genus develop pectoral fins although these fins are absent in adult fish (for *N. lumbriciformis*, see [10]). In *N. ophidion* (this study) pectoral fin buds appear around the onset of eye pigmentation. The blades develop during snout formation, but rays do not form until release; whether they develop at all is questionable. In newborn *Nerophis* spp. (Figure 4A), dorsal and anal fins have only reached the bud stage (cp. Table A1).

Table A1 Fin development in syngnathids

Scientific name	State of fin development in newborn	Types of fins[1] in adult fish	Pouch type[2]	References
Hippocampus spp.	a, d, p: rays	a, d, p	A5	[9,11,12,16], this study
Syngnathus spp.	a: blades (rays about to form)<br>c, d, p: rays	a, c, d, p	A4i	[13,15,36,38], this study
Micrognathus brevirostris	a, p: blades about to form<br>c, d: rays	a, c, d, p	A4e	[34]
Ichthyocampus carce	a: n/a[3]<br>c, d: rays<br>p: blades	a, c, d, p	A4e	[35]
Phyllopteryx taeniolatus	a, d, p: rays	a, d, p	A2	[17]
Microphis leiaspis	a, c, d: buds about to form<br>p: blades	a, c, d, p	B3	[28]
Syngnathoides biaculeatus	a, d, p: rays	a, d, p	B2[4]	[14,39]
Nerophis spp.	a, d: buds<br>p: blades	a, d	B1	[10], this study

[1]a, anal fin; c, caudal fin; d, dorsal fin; p, pectoral fins
[2]A and B pouch types represent tail and trunk brooders, respectively; increasing numbers indicate increasing pouch complexity. A5, sealed pouch; A4i, inverted pouch; A4e, everted pouch; B3, pouch plates; A2 and B2, membranous egg compartments; B1, open brooding area without protective structures (for descriptions of pouch types, see [6,7])
[3]n/a, information not available; adult *I. carce* have two anal-fin rays [37]
[4]Phylogenetically, *S. biaculeatus* clusters with tail-brooding species of the A2-type, suggesting that trunk-brooding in *Syngnathoides* has evolved independently [2]

Additional references

34. James PSBR: ***Micrognathus brevirostris* (Ruppell) (Family Syngnathidae: Pisces) – A new record from the Indian Seas with observations on its early development.** *J Mar Biol Ass India* 1970, **12:**158–162.
35. Jones S, Menon PMG: **Notes on the breeding habits and developmental stages of some estuarine fishes.** *J Zool Soc India* 1953, **5:**255–267.
36. Rao AVP: **A note on the larvae of *Syngnathus cyanospilus* Bleeker from the Pulicat Lake.** *J Mar Biol Ass India* 1968, **10:**398–399.
37. Dawson CE: *Indo-pacific pipefishes (Red Sea to the Americas)*. Ocean Springs: Gulf Coast Research Laboratory; 1985.
38. Silva K, Monteiro NM, Almada VC, Vieira MN: **Development and early life history behaviour of aquarium reared *Syngnathus acus* (Pisces: Syngnathidae).** *J Mar Biol Ass UK* 2006, **86:**1469–1472.
39. Sudarsan D: **On the early development of the pipe-fish *Syngnathoides biaculeatus* (Bloch).** *J Mar Biol Ass India* 1966, **8:**222–224.
